# Internet of Things in Marine Environment Monitoring: A Review

**DOI:** 10.3390/s19071711

**Published:** 2019-04-10

**Authors:** Guobao Xu, Yanjun Shi, Xueyan Sun, Weiming Shen

**Affiliations:** 1 School of Electronics and Information Engineering, Guangdong Ocean University, Zhanjiang 524088, China; xuguobao@126.com; 2 School of Mechanical Engineering, Dalian University of Technology, Dalian 116024, China; syj@dlut.edu.cn; 3 State Key Lab of Digital Manufacturing Equipment and Technology, Huazhong University of Science & Technology, Wuhan 430074, China; sunxueyanxy@163.com

**Keywords:** Internet of Things, Big Data, wireless sensor networks, marine environment monitoring

## Abstract

Marine environment monitoring has attracted more and more attention due to the growing concern about climate change. During the past couple of decades, advanced information and communication technologies have been applied to the development of various marine environment monitoring systems. Among others, the Internet of Things (IoT) has been playing an important role in this area. This paper presents a review of the application of the Internet of Things in the field of marine environment monitoring. New technologies including advanced Big Data analytics and their applications in this area are briefly reviewed. It also discusses key research challenges and opportunities in this area, including the potential application of IoT and Big Data in marine environment protection.

## 1. Introduction

Although the Internet of Things (IoT) has been defined in various perspectives, there is a common version that is widely accepted by scholars as follows: IoT is a dynamic global network infrastructure with self-configuring capabilities based on standard and interoperable communication protocols, where physical and virtual “things” have identities, physical attributes, and virtual personalities and use intelligent interfaces, and are seamlessly integrated into the information network [1]. Recently, IoT has been widely accepted as a promising paradigm that can transform our society and industry. It can achieve the seamless integration of various devices equipped with sensing, identification, processing, communication, actuation, and networking capabilities [2]. A wireless sensor network (WSN) plays a key role in IoT. It consists of a large number of distributed sensors interconnected through wireless links for physical and environmental monitoring purposes. On the other hand, Big Data is considered as an emerging technology and has become a very active research area, primarily involving topics related to data mining, machine learning, database, and distributed computing.

During the past couple of decades, wireless sensor networks (WSNs), as a subset of IoT, have been widely utilized in a variety of smart applications and services, including smart home [3], smart building [4,5], smart transportation [6,7], smart industrial automation [8,9], smart healthcare [10], smart grids [11], and smart cities [12]. Similar IoT-based technologies can certainly be applied to the monitoring and protection of marine environments.

With the development of our society and economy, the marine environment has drawn increasing attention from scientists and scholars. Conventional marine environment monitoring systems such as oceanographic and hydrographic research vessels are very expensive. Their data collection and analysis processes are time-consuming and the collected data have a low resolution. The Internet of Things (IoT) has been evolved from wireless sensor networks (WSNs). Compared with WSNs, IoT has much stronger data processing capabilities, enabling intelligent control of objects.

In a typical IoT-based marine environment monitoring system, different sensors are deployed to measure and monitor various physical and chemical parameters like water temperature and pressure, wind direction and speed, salinity, turbidity, pH, oxygen density, and chlorophyll levels. An advanced IoT-based marine environment monitoring and protection system would also be able to control some objects, devices, or equipment within the monitored marine environment, in order to adjust some physical and chemical parameters so as to improve the marine environment.

While the design, development, and deployment of an IoT-based marine environment monitoring and protection system is needed to address some critical issues including autonomy, adaptability, scalability, simplicity, and self-healing [13,14], following requirements specific to the harsh marine environments should be considered [15]:

(1) High water resistance: Sensor and actuator nodes need to have very high levels of water resistance;
(2) Strong robustness in hardware: Hardware or equipment needs stronger robustness due to the aggressive and complex marine environment with currents, waves, tides, typhoons, etc.;
(3) Low energy consumption and energy harvesting: Energy conservation and harvesting measures need to be considered due to long communication distances and an environment in constant motion;
(4) Stability of Radio signal: Special techniques may be required to ensure the stability of radio signals since the oscillation of the radio antenna can cause an unstable line-of-sight between transmitters and receivers [16] and bad weather conditions can also affect the stability of radio signals;
(5) Other issues: Devices and sensor nodes should be highly reliable because of the difficult deployment and maintenance; the need for buoy and mooring devices; sensor coverage needs to be carefully calculated because of large areas [17]; equipment should be designed against possible acts of vandalism.

We conducted a comprehensive review of WSN applications in marine environment monitoring in 2014 [18]. When we planned and started this updated review, we wanted to expand the scope from WSN to IoT and to cover the protection aspect in addition to the monitoring because of the characteristics of IoT not only on sensing but also on actuation. However, we did not find enough references related to protection. In this paper, we do not consider the protection aspect as a focus but keep the related discussions. We envision the protection from two perspectives: (1) Results from the advanced data analytics based on the data collected by the monitoring system can be fed back to the marine environment management agencies/control centers for quick decision making and real time manual interventions in order to protect the marine environment from some disasters (e.g., oil spills and bad weather damages); (2) some autonomous vessels or other devices (actuators) can also automatically and quickly react to disasters and other events in order to protect the marine environment. Similarly, we wanted to cover the Big Data analytics and its applications to marine environment monitoring and protection, but we did not find sufficient references. Therefore, we did not make the Big Data a focus of this paper. However, we strongly believe that Big Data analytics will play a more and more important role in this area in the future.

The remainder of the paper is organized as follows: Section 2 provides an overview of the fundamentals of IoT-based marine environment monitoring systems; Section 3 presents a summary of some related projects and systems under five application areas; Section 4 reviews a few new technologies applied in the marine environment monitoring systems; Section 5 addresses some research challenges and opportunities in this area; Section 6 provides some concluding remarks.

## 2. Overview of IoT in Marine Environment Monitoring

This section provides an overview of IoT in marine environment monitoring, including various applications, common system architectures, typical sensing nodes and sensing parameters, and related wireless communication technologies.

### 2.1. IoT-based Marine Environment Monitoring Applications

IoT-based marine environment monitoring application areas include: (1) Ocean sensing and monitoring; (2) water quality monitoring; (3) coral reef monitoring; (4) marine (either offshore or deep-sea) fish farm monitoring; (5) wave and current monitoring. Different applications use different IoT system architectures, sensing and control technologies, and communication technologies.

An ocean sensing and monitoring system is a general marine environment monitoring system, which existed for a long time, previously using oceanographic and hydrographic research vessels. A water quality monitoring system usually monitors water conditions and qualities, including water temperature, pH, turbidity, conductivity, and dissolved oxygen (DO) for ocean bays, lakes, rivers, and other water bodies. A coral reef monitoring system typically monitors coral reef habitats and the surrounding environments. A marine fish farm monitoring system monitors water conditions and qualities including temperature and pH, measures the amount of fecal waste and uneaten feed for a fish farm, as well as fish conditions and activities including the number of dead fishes. A wave and current monitoring system measures waves and currents for safe and secure waterway navigations.

### 2.2. Common IoT-based System Architectures for Marine Environment Monitoring and Protection

**Figure 1:**
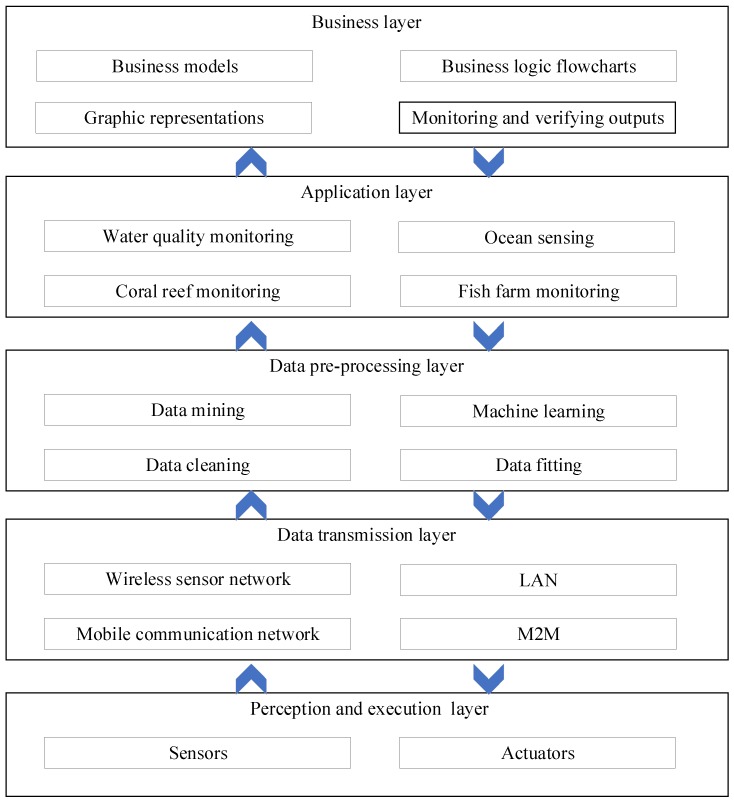
Common layered architecture for Internet of Things (IoT)-based marine environment monitoring and protection applications.

The Internet of Things is usually to achieve “knowing, thinkable, and controllable” to the surrounding world [19], which means that the IoT is able to perceive, think, and control the world by collecting, processing, and analyzing the data of the world. It can make intelligent judgments that impact on the outside world. IoT researchers have proposed different IoT system architectures in the research literature. Among them, a five layered system architecture was proposed by Antao et al. [20]. Similarly, we also believe that a typical IoT-based marine environment monitoring and protection system has five layers: Perception and execution layer, transmission layer, data pre-processing layer, application layer, and business layer, as shown in Figure 1.

(1) Perception and Execution Layer

The perception and execution layer is the bottom layer of the architecture. It includes sensor and actuator devices, with the objective of sensor data collection and command actuation. In IoT-based marine environment monitoring and protection systems, this layer can also include GPS sensors, energy harvesting devices, in addition to regular water condition and quality monitoring sensors. Note that most existing marine environment monitoring systems do not have any execution functions and therefore do not include actuators. 

(2) Data Transmission Layer

The main function of the data transmission layer is to transmit various collected data to the data processing layer via communication networks, mostly mobile or wireless communication networks. At the same time, control measures made by users or intelligent applications (reference engines) are transferred from the application layer to the perception and execution layer, thus that corresponding devices or actuators can take required actions (such as device repositioning, increasing or decreasing temperature settings, releasing food in fish farms).

(3) Data Pre-Processing Layer

The data pre-processing layer is in the middle of the IoT system architecture, where the raw data received can be stored and pre-processed, using advanced data mining technologies. It also completes information aggregation or disaggregation, data cleaning and fitting or screening, sharing as needed, and sometimes it triggers alerts or warnings based on pre-defined rules.

(4) Application Layer

The application layer provides services according to different applications requested by users. For example, it provides water condition and quality data as well as the amount of fecal waste and uneaten feed for a fish farm. The main purpose of this layer is to provide smart application services to meet users’ needs. In IoT-based marine environments, this later covers water quality monitoring, coral reef monitoring, marine (either offshore or deep-sea) fish farm monitoring, wave and current monitoring [21,22,23].

(5) Business Layer

The business layer is the top layer and manages the overall IoT system activities and services, including creating business models, business logic flowcharts, graphic representations, according to the data received from the application layer. It also monitors and verifies outputs of the other four layers according to the business models in order to enhance services and maintain users’ privacy [21,23].

**Figure 2:**
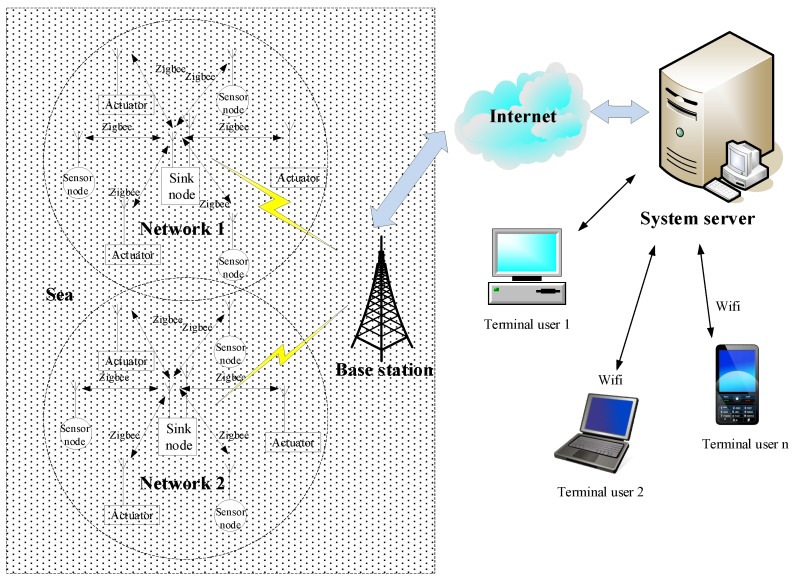
Common physical architecture of IoT-based marine environment monitoring and protection systems.

Such a layered system architecture provides a good picture of the data/information flow in IoT-based marine environment monitoring and protection systems. Figure 2 shows a common physical architecture of IoT-based marine environment monitoring and protection systems. It includes sensor nodes and actuator nodes, sink nodes, a base station, a system server, and user terminals. Sensor nodes are used to sense and monitor environmental parameters such as water temperature and pH, salinity, turbidity, oxygen density, and chlorophyll levels, and transmit the collected data to sink nodes via ZigBee or some other wireless communication protocols. Actuator nodes execute commands from the upper layers. Communication between a sink node and sensor or actuator nodes is usually point-to-point. A sink node collects data from a group of sensor nodes and sends collected data to the base station or passes execution commands from upper layers to actuator notes, via mobile communication networks (2G/3G/4G). The base station is connected to a system server through the Internet. The system server stores and processes the received data from the base station, completes data analyses according to corresponding applications, sends commands to actuator nodes according to the pre-defined rules as appropriate. Various kinds of user terminals (desktops, laptops, pads, and smart phones, etc.) connect users to the system server over the Internet (including mobile Internet).

The design and development of a durable and scalable IoT system for marine environment monitoring and protection should carefully consider a number of factors: The harsh marine environment, the communication protocols and network topology, the number and distribution of nodes, buoys and mooring systems, oceanographic sensors, energy supply and harvesting options, and so on.

**Figure 3:**
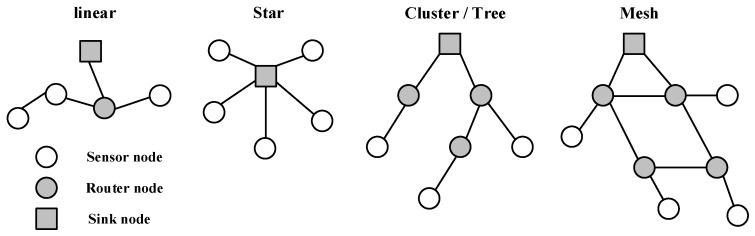
Typical wireless sensor network topologies.

As described above, an IoT system consists of many sensor and actuator nodes and a gateway for the connection to the Internet. Usually, sensor and actuator nodes are organized into a connected network according to a certain topology. Physical topology and density are entirely dependent on applications [24], therefore the design and development of an IoT system need to consider its application and deployment environment. Even though more sensors can be densely deployed to enhance data availability and accuracy, a dense deployment also brings negative issues: High energy consumption, data collisions, and interferences, etc. [25]. Sensor nodes normally have four typical kinds of network topologies: Linear, star, cluster/tree, and mesh topologies, as shown in Figure 3. While we have discussed in detail the star, cluster/tree, and mesh topologies in reference [18], the linear topology is a series connection of all the nodes. Its main advantage is that it has a simple structure thus that the cost is relatively low. As a consequence, the transmission volume is large, and the speed is slow.

The selection of a right network topology for a particular application depends on the amount and frequency of data to be transmitted, the distance of data transmission, the requirement of battery life as required for maintenance, and the mobility of the sensor nodes [26]. On the other hand, the physical network topology of an IoT system may change during its operation due to its energy availability, node position variations, equipment or sensor malfunction, node reachability (due to noise, severe weathers, moving obstacles, etc.), and task details of sensor nodes [27].

### 2.3. A General Marine Environment Monitoring Sensor Node

**Figure 4:**
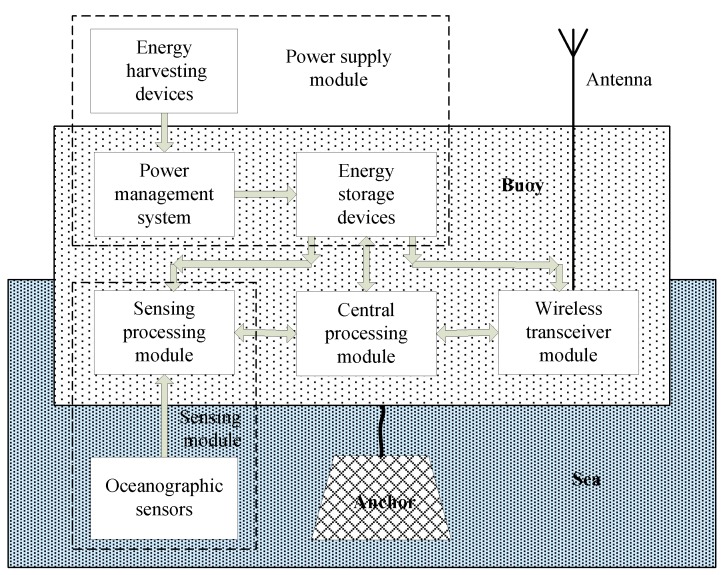
General architecture of a marine environment monitoring sensor node.

The general architecture of a marine environment monitoring sensor node is shown in Figure 4. It typically has a buoy device to protect electronic devices against water, and consists of the following four main modules [28]: A sensing module, a microcontroller, a wireless transceiver module, and a power supply module.

The sensing module obtains the environmental and equipment status (with associated amplifiers and analog-to-digital (A/D) converters). The microcontroller receives the data from the sensor and processes the data accordingly. A wireless transceiver module includes a radio frequency (RF) transceiver and an antenna. A power supply module includes energy storage devices (like rechargeable batteries), and a power management system with energy harvesting devices, which can be a solar panel, a wind energy harvesting device, a tidal power generator, or a seawater power generator. The buoy has an anchor to prevent it from moving.

### 2.4. Typical Sensors and Sensing Parameters

Sensors are used to respond to changes in their environments by producing electrical signals in the form of electrical voltage, current, or frequency [29]. There are typically two kinds of sensors: Physical sensors and chemical sensors. Physical sensors are used to measure different physical parameters like temperature, humidity, pressure, wind speed, and wind direction. Chemical sensors are used to measure various chemical parameters like salinity, turbidity, pH, nitrate, chlorophyll, and dissolved oxygen (DO). Details can be found in reference [18]. Decisions on the selection of sensors are made according to requirements related to the deployment area and season, measurement range, accuracy, resolution, and power consumption.

### 2.5. Wireless Communication Technologies

A sensor node requires a radio module for wireless communication. The access network, with a communication range from a few hundred meters to several kilometers, includes all the devices between the backbone network and user terminals.

For the IoT-based marine environment monitoring and protection systems, wireless communication networks have different requirements than other applications, because of the following reasons:

(1) Reliability: Radio antenna oscillations and bad ocean weather conditions can cause instability of radio signals.
(2) Energy efficiency: Low power consumption is the key to supporting long-flow and reduced maintenance costs in stand-alone battery-powered equipment. This is particularly critical for devices deployed in remote offshore areas that are difficult and costly to replace.

According to the current requirements for IoT applications, the development of wireless communication technologies has already made significant progresses. Various wireless communication standards and technologies have been proposed and developed, including WiFi, ZigBee, Bluetooth, GPRS, GSM, and WiMAX. A summary and brief comparison of these communication standards and technologies can be found be in our previous survey paper [18]. Typically, multiple wireless communication technologies are used in an IoT-based marine environment monitoring and protection system. In some specific applications, underwater acoustic communication technologies are used for data collection and communication among underwater marine environment sensors [30,31,32,33]. Generally, a longer-range communication consumes more energy. Selection of the most appropriate wireless communication technology for an application depends on the transmitted data volume, transmission frequency, transmission distance, and available power supply. 

## 3. A Review of Existing Marine Environment Monitoring Projects and Systems

Various systems have been developed and deployed for marine environment monitoring during the past couple of decades. Table 1 summarizes the features of reviewed projects and systems under five different application areas as defined in Section 2.1. For completeness and consistency, we kept all the projects and systems summarized in our previous survey [18] and added more from the recent research literature.

From this long list of systems, we can see that most of the efforts are related to general ocean sensing and monitoring [34,35,36,37,38,39,40,41,42,43,44,46,47,48] and water quality monitoring [53,54,55,56,57,58,59,60,62]. Some specific efforts have been made for fish farm monitoring [63,65,66,68], coral reef monitoring [61,62], wave and current monitoring [69], and marine shellfish monitoring [64]. Several projects focus on specific technologies or devices, e.g., buoys [35,36,44,45,47,64], energy saving and harvesting [34,40,43,44,45,55,59,63,64], routing protocols [46,64], data transmitting approaches [38,46,47], and data analysis [62].

It can also be found that testing places are different among these systems. About half of the developed systems have been tested or deployed in real marine or river environments [34,35,37,39,40,41,44,45,46,47,55,56,57,60,61,65,69]; a number of them were experimented in lab settings or indoor environments [38,53,64,68]; some were tested in outdoor pools or small ponds/lakes [36,54,59,63]; and several of them were only tested through simulations [38,66,68].

It is very interesting to note that most projects, systems, and applications have been developed by research groups in a small number of countries, including China [37,41,42,58,64], USA [36,53,54,61], Spain [38,45,63,68], UK [46], Indonesia [62], Philippines [69], India [48], Portugal [47], France [65], and Ireland [55,56]. In these completed systems, most of them occurred on water surfaces [34,35,37,39,40,41,42,43,44,45,46,48,54,55,56,57,58,59,60,61,62,63,64,65,68,69] and only a few of them were deployed under water [38,47,53,66]. Please note that underwater wireless sensor network is not the focus of this paper and we will briefly discuss this later in the paper. 

Even though many researchers discussed different options for energy harvesting including solar, wind, waves, and ocean currents, only solar energy has been used in about a third of these systems and applications.

While most systems use GPRS and/or ZigBee for wireless communications, a few systems use underwater acoustic communication [38,47,53,66].

Offshore and deep-sea fish farming has been emerging, which provides more opportunities and also challenges, not only for marine environment monitoring but also for operation controls and marine environment protection.

## 4. New Technologies for Marine Environment Monitoring and Protection

### 4.1. Data Analysis

Recent fast development and deployment of IoT technologies in marine environment monitoring created huge amounts of data, while the recent advancement of Big Data analytics facilitated the analysis of these marine environment data. 

As in many other IoT-based data collection systems, dealing with marine environment data also faces some major challenges, particularly the large amount of data and significant bad data. Researchers around the word have been trying to address these challenges. Yang et al. [71] proposed a method to quickly describe the contour of data collected over the Internet of Things (IoT). The distribution of contour lines can be calculated accurately in a short time. 

Blix and Eltoft [72] proposed an automatic model selection algorithm (AMSA) to determine the best model for a given matchup dataset. It can automatically choose between regression models to estimate the parameter of interest. It also finds out the number and combination of features to be used for obtaining the best model. They used four Machine Learning feature ranking methods and three Machine Learning regression models to estimate oceanic chlorophyll-a in the global and optically complex waters.

Zhong et al. [73] developed a fast fuzzy C-means clustering algorithm to analyze water environment monitoring data of the Three Gorges Reservoir Area. The hard cluster center can be treated as the initial value of the fuzzy cluster center to accelerate the speed of convergence and reduce the number of iterations.

Addison et al. [74] summarized the challenges in marine environment data management and interpretation caused by the implications of Big Data. They suggested a solution for the management of Big Data, which requires new collaborations between marine practitioners and data scientists with expertise in programming languages and packages like R and Python.

Belghith et al. [75] proposed a deep learning-based approach in a marine Big Data setting that enables to classify these diverse acoustic sounds not only considering marine mammals signals.

Li et al. [76] presented a support vector regression architecture with smoothness priority for marine sensor data prediction to handle the abruptly fluctuating, multi-noise, non-stationary and abnormal data. The smoother plays the role of preprocessing to handle the outliers and noises in marine sensor data, providing stable initialization values for the next nonlinear approximation based on support vector machines.

Since the existing current correlation analysis method for ocean monitoring big data is time consuming and stability is poor, Song et al. [77] developed a new method by sending the collected data to the cloud storage system. Based on the global and local Moran index calculations, the ocean big data of relatively high correlation were saved to the adjacent data center, which reduces the ocean monitoring data correlation analysis time.

Radeta et al. [78] developed a low-cost passive acoustic monitoring (PAM) system for nautical citizen science and real-time acoustic augmentation of whale-watching experiences. This paper used machine learning identify vocal acoustic samples of common cetaceans like whales and dolphins with acoustic features (clicks, moans or whistles).

It is clear that research and development efforts on the application of Big Data analytics in marine environment monitoring have been growing recently.

### 4.2. Network Topology Control

In wireless sensor networks (WSNs), network topology control capability is a key factor in the performance of the entire network. A reasonable WSN topology control structure can effectively improve the efficiency of network communication protocols and the overall performance of the network. A good network topology helps extend the overall life cycle of the network. Therefore, WSN topology control optimization technology is the key to determining the overall performance of the network, including the network coverage and connectivity. The node selection policy changes the state of the node itself and avoids the communication link redundancy among nodes to form a performance-optimized network structure. Table 2 summarizes the advantages and disadvantages of different topology control algorithms for underwater wireless senor networks (UWSNs) [79]. 

Even though this classification of topology control algorithms is for underwater wireless sensor networks, it can be well applied to the IoT-based marine environment monitoring in general. Please note that UWSN is not the focus of this paper. For comprehensive reviews of underwater sensor networks and applications, please refer to references [80,81]. It is also interesting to check out the concept of Internet of Underwater Things (IoUT) and its potential applications [82].

The rest of this section provides a review of some examples of interesting approaches proposed and developed in the literature on network topology control. Kim et al. [83] used power control to achieve reliable data delivery based on the sea surface movement that affects the surface signal reflection and the strength of the received signal at a node. Bai et al. [84] proposed an approach to reduce link interference and achieved high throughput by using a correlation matrix to describe the source-destination relationship and conflict relationships among the links. Power control is used to achieve a Signal to Noise Ratio (SNR) larger than the decoding threshold. 

Su et al. [85] proposed a cycle difference set-based protocol to determine the number and positions of active and sleep intervals in one cycle to guarantee that both the transmitter and receiver are awake for communication. Coutinho et al. [86] developed an optimization model to investigate the performance of the on-the-fly adjustment of the sleep interval in duty-cycled UWSNs to achieve a balanced energy consumption.

Khan et al. [87] presented a method to incorporate the AUV resurfacing time in the VoI function and AUV path planning algorithm. Coutinho et al. [88] designed the distributed topology control (DTC) and centralized topology control (CTC) depth adjustment–based topology control algorithms for disconnected and void nodes repositioning to improve network connectivity and data routing.

### 4.3. New Communication Routing Protocols

With the advancement of communication technologies, new communication routing protocols have been proposed and developed for marine environment monitoring systems. Faheem et al. [89] proposed a chromosome (routing path), which consists of sequences of non-negativity integers that denote the IDs of genes (CH nodes) through which a routing path passes. In a routing path, the order of each CH is represented by the locus of chromosome in which the gene of the first locus is always reserved for the source CH nodes. A looping feature of selection (φs), crossover (φc), and mutation (φm) operators is applied on each individual to improve the quality of the solution through the pre-defined probabilities (φp) until the termination criterion is satisfied. The probability of the mutation rate increases from an extremely low value of 0.01 to its maximum value of 0.05. Highly stable small clustering mechanism is used to organize sensor nodes into a connected hierarchy for distributing energy and data traffic load evenly in the network.

The protocol proposed by Javaid et al. [90] is called balanced energy adaptive routing (BEAR). It exploits the location information, selects the neighbors, chooses the facilitating and successor nodes based on cost function value, and finally selects the forwarder node that has residual energy more than the average residual energy of the network. BEAR allows nodes to communicate directly with the sink node leading to an increase in the number of packets being dropped.

Alageswaran and Swapna [91] proposed an enhanced duty cycled multiple rendezvous multichannel media access control (DMM-MAC) for handling more volume of data in multi-hop underwater wireless sensor networks (UWSNs) for marine eco systems. This protocol distributes bursty sensor data by dynamically tuning the duty cycles, and hence network performance is enhanced dramatically. It is equipped with one modem and the propagation delays or relative distances need not to be known by the other nodes in the network. Less energy is consumed, and it is easy to forward the data based on priority. One cycle consists of several frames and is subdivided into an active section and a sleep section. Each node can use the other time slot when the time slot is free.

## 5. Major Challenges and Opportunities

### 5.1. Energy Management

Because marine environment monitoring and protection systems work on hash environments as mentioned above, battery replacement is difficult and costly. It is therefore very important to have an efficient energy management system together and preferably with an energy harvesting capability.

Typical energy options for sensor nodes include batteries, capacitors, fuel cells, and energy harvesting. A battery is widely used in sensor nodes, and in fact in over half of the systems reviewed under this paper. However, using batteries in sensor nodes has a number of issues [92] including the replacement difficulty, risk of losing power during operation, and environment contamination. It is therefore important to explore alternative power supply options for sensor nodes. Energy harvesting is a natural way to go. In marine environments, energy harvesting options including solar, wind, water waves, and currents. The most outstanding energy harvesting at the moment is photovoltaics (solar energy) [92]. 

Usually, network energy consumption is increasing at a very high rate due to an increase in data rates, increase in the number of Internet-enabled services and rapid growth of Internet connected edge-devices [21]. The drastic increase in IoT devices requires efficient fabrication of batteries because of the uncertainty in battery dissipation. On the other hand, the devices in marine environment monitoring and protection systems are heterogeneous in nature, each with various capabilities and numerous requirements [93]. Hence, the requirement of cost effective and energy efficient routing strategy in terms of space and time arises in future wireless communication networks.

### 5.2. Standardization

With the wider scope of marine environment monitoring and protection, international cooperation projects are emerging one after another. According to the interests of the projects, various application platforms and methods have their own characteristics and cannot be compatible with each other. Even though a number of networking protocols and standards for Internet of Things have been proposed and developed [94,95], they are not sufficient for applications in marine environment monitoring. In addition to the standards of IoT networking, it is also important to provide the industry with standards for IoT devices, equipment, and platforms for marine environment monitoring and protection applications, and providing different levels of governments and marine environment management agencies with standards for marine environment data management, analysis, and reporting. As a result, the standardization of platform development for marine environment monitoring and protection systems brings challenges, including:

1. Standardization of IoT devices specifically for marine environment monitoring and protection, including sensor and actuator nodes, routers and gateways.
2. Standardization of IoT platforms and system technologies for marine environment monitoring and protection, including communication network structures, protocols, and algorithms.
3. Standardization of computing and data storage technologies for marine environment monitoring and protection, including cloud, fog, and edge computing mechanisms, data archiving and warehousing techniques.
4. Standardization of data analysis outputs and reporting formats for exchange among different organizations and governments.

### 5.3. Marine Environment Protection

Currently, the environmental protection issue is one of the most important issues around the world. The ultimate goal of monitoring the ocean is to protect the marine environment. Most of the current marine environment monitoring applications collected and analyzed massive data from the ocean, but there is no action on protection controls yet. With the advancement and sophistication of IoT and Big Data technologies and their wide applications in marine environment monitoring and protection, we are confident that active marine environment protection measures, technologies, and systems will be developed and deployed in the near future. Massive data collected from marine environments will be analyzed using advanced Big Data analytics and the results will be sent to the related marine environment management agencies/control centers for quick decision making and real time manual interventions in order to protect the marine environment from some disasters (e.g., oil spills and bad weather). On the other hand, some autonomous vessels or other devices (actuators) can also automatically and quickly react to disasters and other events in order to protect the marine environment. In the case of a fishing farm application, data collected from the fish farm environment can be analyzed and used to provide the best fish growth condition and the best feeding. 

## 6. Conclusions

During the past couple of decades, marine environment monitoring has attracted wide attention. Governments and research organizations have heavily invested in the research and development of new technologies in this area. Advanced information and communication technologies have been applied to the development of various marine environment monitoring technologies and systems. Internet of Things has played an important role in this area. This paper presents an updated review of the related technologies and systems on the application of the Internet of Things in marine environment monitoring.

A comprehensive review of about 40 related projects revealed that most systems and applications developed thus far are for ocean sensing and monitoring and water quality monitoring. Some specific efforts have been made for fish farm monitoring, coral reef monitoring, wave and current monitoring. Several projects focused on specific technologies or devices like buoys, energy saving and harvesting devices, routing protocols, data transmitting mechanisms, and data analysis techniques.

It can also be noted that testing places are very much different among these projects. About half of the developed systems have been tested or deployed in real marine or river environments; a number of them were experimented in lab settings or indoor environments; a few others were tested in outdoor pools or small ponds and lakes; and several of them were only tested through simulations.

While most systems use GPRS and/or ZigBee for wireless communication, a few systems used underwater acoustic communication.

Emerging offshore and deep-sea fish farming provides more opportunities and also challenges, not only for marine environment monitoring but also for operation controls and marine environment protection, which is a new area of research.

The energy management issue is mainly considered from two aspects: Reducing energy consumption and using alternative renewable energy sources. Optimizing network topologies and developing advanced routing protocols are the ways to reduce energy consumption. Even though many researchers discussed different options for energy harvesting including solar, wind, waves and ocean currents as alternative renewable energy sources, only solar energy has been used in about a third of the developed systems. 

The results of Big Data analytics can be used not only for feedback to marine environment management agencies and control centers for quick decision making and real time manual interventions but also for autonomous vessels and remotely deployed devices to take real time actions in order to protect the marine environment from some disasters (e.g., oil spills and bad weather). It is a growing area of research and development.

This review also identified several research challenges and opportunities, including energy management, standardization of system platforms and technologies, and marine environment protection measures.

## Figures and Tables

**Table 1:** Summary of existing marine environment monitoring projects and systems.

Reference	Country	Sensing Parameters	Comm. Protocols	Buoy	Energy Harvesting	Key Features (Including Testing and Deployment)
**Application Area: Ocean Sensing and Monitoring**						
Perez et al. [34]	Spain	T, P, salinity, nitrates, velocity, chlorophyll, and turbidity	GPRS, ZigBee	Special buoy	Solar	LabVIEW-based user interface using Google Maps; Solar energy harvesting; Special buoy; Deployed in a harbor
Voigt et al. [35]	Sweden; Germany	T, motion, vibration and sound	GPRS	Simple buoy and king’s buoy	No	Design of an advanced low-cost buoy system; tested in real environment
Vesecky et al. [36]	USA	T, wave and location	900 MHz	Mobile minibuoy	No	An autonomous mini-buoy prototype (tested in a pool); GPS is used
Liu et al. [37]	China	T, Sea depth	ZigBee	Sensor floating	No	A Perpendicular Intersection (PI) mobile-assisted localization scheme; deployed in Hong Kong U of S&T campus and Tsingtao
Macias et al. [38]	Spain	T, Visible-field, sound	ZigBee and acoustic	?	?	Three-tier communication architecture; transmitting video streaming data; Tested on module of NS-3
Roadknight et al. [39]	UK	T, conductivity, water depth, turbidity	?	Single buoy	No	A multi-layered scalable and adaptive approach of data management; deployed off Scroby sands
Cella et al. [40]	Australia	T, illuminance	ZigBee	Cylinder waterproof buoys	Solar	Used underwater wireless communication; deployed in the Moreton Bay
Jiang et al. [41]	China	T, velocity and light	ZigBee	Lever buoy	No	The sleep mechanism and lever buoy; deployed off the seashore
Tao et al. [42]	China	Water T, DO and pH	ZigBee	Buoys with GPS and PEA	?	Position determination and location verification using GPS and PEA (positioning estimation algorithms); tested in two testbeds
Alippi et al. [43]	Italy	Seawater luminosity, T and moisture	ZigBee	Cylinder waterproof buoys	Solar	Optimal solar energy harvesting; power-aware and adaptive TDMA protocol; deployed in the Moreton Bay
De Marziani et al. [44]	Argentina	T, P, PAR radiation, pH and salinity	ZigBee	Cylinder waterproof buoys	Solar	A low cost reconfigurable WSN with solar panels; tested in San Jorge Gulf
Albaladejo et al. [45]	Spain	T, P	ZigBee	Special buoy	Solar	A new multisensory buoy system with solar panels; deployed in Mar Menor Lagoon
Al-Zaidi et al. [46]	UK	T, depth, wind speed and direction, humidity, salinity	MADNET routing protocol	Ship	?	Marine data acquisition and cartography system based on VHF; hybrid Mobile Ad-hoc/Delay Tolerant routing protocol (MADNET); tested in North Sea, and English Channel
Ferreira et al. [47]	Portugal	T, position	WiFi, GPRS/UMTS/LTE, Acoustic	Ship Buoy, ASV	No	Used autonomous underwater vehicles (AUV), and autonomous surface vehicles (ASV); tested in Portuguese coast
Kaur et al. [48]	India	Water T, P, wind speed, wind direction, humidity, cloud cover, turbidity	GPRS		?	SentiWordNet is used as an information retrieval tool for processing messages received from nearby marine areas
Hu et al. [49]	China	T, humility and salinity	?	AUV	?	Ring Broadcast Mechanism is used to guide searching direction of sensor nodes; providing self-adaptive dynamic routing mechanism to search the alternative path
Mourya et al. [50]	UK	T, P, salinity, oxygen level	Acoustic	Anchors with acoustic modems	Solar	A framework for spatio-temporal monitoring of underwater acoustic sensor networks; anchors are deployed in the ROI inspired by compressive sensing
Morozs et al. [51]	UK	T, P, humidity, optical, distance, sound, magnetic field, motion	Acoustic	Autonomous surface vehicle (ASV)	No	Implementation of the TDA-MAC protocol in practice, and practical issues prompted several crucial modifications to the TDA-MAC protocol
Song et al. [52]	China	Water T, P, salinity and PH	Acoustic	Buoy	?	Underwater positioning algorithm of electing anchor nodes and the self-repairing localization algorithm based on anchor nodes failure
**Application Area: Water Quality Monitoring**						
Yang et al. [53]	USA	pH	RF and acoustic	PVC housing	No	Various interface circuits; 5 air-based sensor nodes; lab testing only
Seders et al. [54]	USA	T, pH, and DO	433 MHz	Box and polyethylene ring	No	A LakeNet sensor pod and an altered sampling strategy; tested a prototype in a small lake
Regan et al. [55]	Ireland	T, pH, turbidity, DO and conductivity	ZigBee	Inshore sensor buoys	Solar	A real-time heterogeneous water quality monitoring; deployed in five sites on the River Lee, Ireland
O’Connor et al. [56]	Ireland	T, conductivity and depth	?	Buoys	?	A multi-modal environment monitoring network based on WSN and visual image; tested in River Lee, Poolbeg Marina and Galway Bay
Hadjimitsis et al. [57]	Cyprus	T, P, salinity and turbidity	GPRS	Cylinder waterproof buoy	No	Integrated satellite remote sensing and WSN; deployed in a beach
Jin et al. [58]	China	T, pH, DO, and salinity	ZigBee GPRS	?	No	An early WSN-based water monitoring system
Alkandari et al. [59]	Kuwait	Water T, DO, and pH	ZigBee 802.11 Ethernet radio	?	Solar	Used ZigBee and 802.11 and a high capacity solar panel; tested in a pool
Adamo et al. [60]	Italy	T, salinity, conductivity, turbidity and chlorophyll-a	GPRS	Self-sufficient buoy	?	Two different probe solutions for field covering; tested in Apulia region
**Application Area: Coral Reefs Monitoring**						
Bromage et al. [61]	USA	T, P, pH, light, and conductivity	900 MHz	Watertight housing	No	Deployed in Monterey Bay
Berlian et al. [62]	Indonesia	T, ORP, pH, Electrical Conductivity, DO, audio/video	?	Remotely Operated Vehicle, buoy	No	Remotely Operated Vehicles with water quality sensors; Big Data analysis
**Application Area: Fish Farm Monitoring**						
López et al. [63]	Spain	T and pH	ZigBee	?	No	A sub-layer-based power consumption algorithm; tested in a pool
Yang et al. [64]	China	Water T, pH value, salinity, DO and COD	GPRS	?	Solar	Multi-hop communication protocol, multiple nodes, and SMT; tested in an aquatic experimental base
Leblond et al. [65]	France	T, depth, salinity, position, catches	GPRS	Vessels	No	Fixed on fishing gears, self-powered, autonomous; tested in Bay of Biscay
Lloret et al. [66]	Spain	Sediment depositions	Acoustic	Bouy	?	Ultrasonic sensor; tested through simulations
Meera et al. [67]	India	Sea surface T, quality of sea water, pH, chlorophyll	WiFi	Fishing vessels	No	A multi-level P2MP infrastructure network——OceanNet; protocol performance comparison of CoAP, AMQP and MQTT
Lloret et al. [68]	Spain	Amount of pollution	?	Buoy	?	A group-based underwater WSN for monitoring fecal waste and uneaten feed; tested on OPNET Modeler network simulator
**Application Area: Wave and current monitoring**						
Marimon et al. [69]	Philippines	Acceleration, angle	ZigBee, GSM/GPRS/EDGE	Buoy	Solar	Integrated different wave sensors; threshold values generated based on statistics; tested in Manila Bay
Chen et al. [70]	China	Current velocities	?	?	No	A temporal evolution model to describe the ocean current process based on the temporal correlation of the current velocity.

Notes: “T”: Temperature; “P”: Pressure; “DO”: Dissolved Oxygen; “COD”: Chemical Oxygen Demand; “No” under Energy Harvesting: Battery power is used; “?”: Related information is not available from the source.

**Table 2:** Classification of Topology Control Algorithms for underwater wireless senor networks (UWSNs).

Category	Main Idea	Advantages	Disadvantages
Power control based	The proper transmission power level is assigned to each node to guarantee enough signal strength at the receiver that it can successfully receive and decode the transmitted message	Simple; scalable; conserves energy; does not change the sensing coverage; can overcome time-varying acoustic channel quality.	May diminish the network connectivity; increases the number of hops and end-to-end delay.
Wireless interface mode management based	The wireless interface of nodes alternates between active, sleeping, and powered-off modes. This change reduces the amount of unnecessary time a node spends listening to the channel.	Simple; scalable; conserves energy relative channel polling; does not change sensing coverage.	Changes network density; changes routing paths from time to time; increases delay.
Mobility assisted based	Some mobile nodes are moved to new locations in different depths or with a predetermined trajectory, creating new interconnections.	Improves network connectivity; deals with network partitions; improves data collection from hop spots.	Needs trajectory planning procedures; increases energy cost for mobility; may change sensing coverage.
